# Dual Convolutional Neural Network Based Method for Predicting Disease-Related miRNAs

**DOI:** 10.3390/ijms19123732

**Published:** 2018-11-23

**Authors:** Ping Xuan, Yihua Dong, Yahong Guo, Tiangang Zhang, Yong Liu

**Affiliations:** 1 School of Computer Science and Technology, Heilongjiang University, Harbin 150080, China; xuanping@hlju.edu.cn (P.X.); yihua_dong_hlju@163.com (Y.D.); yongliuhlju@gmail.com (Y.L.); 2 School of Information Science and Technology, Heilongjiang University, Harbin 150080, China; guoyahong_hlju@163.com; 3 School of Mathematical Science, Heilongjiang University, Harbin 150080, China

**Keywords:** miRNA–disease association, convolutional neural network, random walk, network topology structure

## Abstract

Identification of disease-related microRNAs (disease miRNAs) is helpful for understanding and exploring the etiology and pathogenesis of diseases. Most of recent methods predict disease miRNAs by integrating the similarities and associations of miRNAs and diseases. However, these methods fail to learn the deep features of the miRNA similarities, the disease similarities, and the miRNA–disease associations. We propose a dual convolutional neural network-based method for predicting candidate disease miRNAs and refer to it as CNNDMP. CNNDMP not only exploits the similarities and associations of miRNAs and diseases, but also captures the topology structures of the miRNA and disease networks. An embedding layer is constructed by combining the biological premises about the miRNA–disease associations. A new framework based on the dual convolutional neural network is presented for extracting the deep feature representation of associations. The left part of the framework focuses on integrating the original similarities and associations of miRNAs and diseases. The novel miRNA and disease similarities which contain the topology structures are obtained by random walks on the miRNA and disease networks, and their deep features are learned by the right part of the framework. CNNDMP achieves the superior prediction performance than several state-of-the-art methods during the cross-validation process. Case studies on breast cancer, colorectal cancer and lung cancer further demonstrate CNNDMP’s powerful ability of discovering potential disease miRNAs.

## 1. Introduction

miRNAs are non-coding single-stranded RNA molecules encoded by endogenous genes with a length of about 22 nucleotides. miRNAs exert their biological functions primarily via regulating the expression of target genes (mRNAs). miRNAs usually target to a specific sequence in the 3′ untranslated terminal of mRNAs, inhibiting the translation of the target genes [1,2,3,4,5]. With the development of molecular biology and biotechnology, scientists find that the abnormal expression of miRNAs is closely related to various human diseases [6,7,8]. Therefore, predicting the potential disease-associated miRNAs is of great significance for understanding disease etiology and pathogenesis.

In recent years, several computational methods have been proposed for predicting disease-associated miRNAs, which can be classified into two main categories in general. miRNAs implement their biological functions by regulating the expression of their target mRNAs [9]. Therefore, the first category of methods is based on target genes to predict the potential associations between diseases and miRNAs. Jiang et al. [10] estimated the functional similarities of miRNAs through the number of target genes co-associated with miRNAs. The similarities among diseases is measured according to the phenotype of the disease, and the known miRNA–disease associations are combined to predict the potential miRNA–disease associations. However, the number of experimentally validated target genes is not sufficient, which cannot provide sufficient and effective data to support the prediction. Li et al. [11] used target gene prediction software TargetScan [12], MiRanda [13], and PITA [14] to predict target genes that a certain miRNA might regulate. The disease-related miRNAs are then predicted by measuring the functional consistency between the predicted target genes and existing disease-related genes. As the false-positive rate of target genes predicted by the software are very high, it is difficult for this method to achieve high prediction accuracy. The methods in the second category are based on the biological observation that miRNAs with similar functions are usually associated with similar diseases and vice versa [15,16,17,18]. Xuan et al. [19] and Xiao et al. [20] proposed the method based on non-negative matrix factorization from the similarity and association perspective of miRNAs and diseases. Liu et al. [21] and Liao et al. [22] proposed the method of predicting miRNA–disease associations via random walking in networks composed of multiple data sources. Zeng et al. [23] proposed a disease miRNA prediction algorithm based on the structural perturbation method. Chen et al. [24] and Zhang et al. [25] proposed a path-based method for predicting miRNAs that are associated with diseases. Ding et al. [26] integrated known miRNA–disease associations and experimentally validated miRNA–target associations and proposed a prediction method based on a disease–miRNA–target heterogeneous network. As these methods are based on the traditional computing model [27,28,29], it is difficult to extract the deep feature representation from the multiple kinds of data.

There are limited associations between miRNAs and diseases, so their associations are sparse. The similarities between diseases are also sparse. Since convolutional neural networks (CNNs) are suitable for dealing with this kind of sparse data [30], we propose a CNN-based prediction method. The topological structures of miRNAs and diseases are also very important for miRNA–disease association prediction. Therefore, we construct a dual CNN-based prediction model to learn the depth feature representation in sparse data and capture the topological information in miRNA and disease networks.

## 2. Results and Discussion

### 2.1. Performance Evaluation Metrics

Considering that most of the diseases in the HMDD database are only associated with a few miRNAs, they are not sufficient to evaluate the prediction performance of our method. Therefore, we performed five-fold cross-validation on the 15 diseases associated with more than 90 miRNAs to compare the prediction performance between CNNDMP and several state-of-the-art methods. First, we regard the known miRNA–disease associations as positive samples, and randomly divide them into five equal parts, and the unknown associations are regarded as negative samples. The negative samples (whose quantity is equal to that of the positive samples) are selected randomly from all the negative ones. These negative samples are also divided into five equal parts. Four parts of positive samples and four parts of negative samples are used as the training data in each-fold cross-validation. The remaining positive and the remaining negative samples are used as the testing data to verify the prediction performance.

We can obtain the association prediction scores in the testing data via the CNN prediction model and sort them by their values in descending order. If a known association exists between a pair of miRNA–disease sample, and the prediction score of the association is higher than the given threshold δ, it is a successfully identified positive sample. If the prediction score of a negative sample is lower than δ, it is a successfully identified negative sample. By changing the threshold, we can calculate the corresponding true positive rate (TPR), false positive rate (FPR), precision (Precision) and recall rate (Recall). They are defined as follows,

(1) $$TPR=\frac{TP}{TP+FN},FPR=\frac{FP}{TP+FP}$$

(2) $$Precision=\frac{TP}{TP+FP},Recall=\frac{TP}{TP+FN}$$

where *TP* and *TN* represent the number of positive and negative samples correctly identified, *FP* represents the number of negative samples misidentified as positive samples, and *FN* represents the number of positive samples misidentified as negative samples. Each time the threshold δ is changed, the corresponding *TPR* and *FPR* values, as well as the *Precision* and *Recall* values, are obtained. The receiver operating feature curve (ROC) and the precision–recall curve (PR) are then drawn using these values. The areas under the ROC curve (ROC-AUC) and the PR curve (PR-AUC) are used to evaluate the whole prediction performance.

Biologists usually select the top-ranked miRNA candidates from the prediction result to further validate their associations with the disease. Therefore, we calculate the average recall values of the top 30, 60, 90–210 and 240 candidates for 15 diseases. Through the recall, we compare how many positive samples appear in the top *k* candidates in different methods. The larger the recall value, the more positive samples are identified successfully.

### 2.2. Comparison with Other Methods

CNNDMP is compared with GSTRW [22], DMPred [19], PBMDA [24] and Liu’s Method [21], which are state-of-the-art prediction methods for miRNA–disease associations. The parameters involved in each method need to be adjusted to achieve the best prediction performance. In our method, $w_{f}$, $w_{p}$ and d are set to 3, 2 and 11, respectively. Each convolutional layer contains 20 convolution filters, so $n_{conv}$ is set to 20. The restart probability β of random walk is 0.8, and the harmonic parameter λ is set to 0.9. λ varies from 0.1 to 0.9, and the corresponding performances of CNNDMP are listed in Table 1. For the other methods, we use the parameters mentioned in the corresponding papers (γ=θ=0.2,α=β=0.8,λ=η=0.2,w=0.6 for GSTRW, L=3,α=2.26 for PBMDA, $\lambda_{M}=\frac{1}{70},\lambda_{D}=\frac{1}{10},\theta =\frac{1}{20}$ for DMPred, λ=0.8,δ=0.9,η=0.1,γ=0.5 for Liu’s Method).

**Figure 1:**
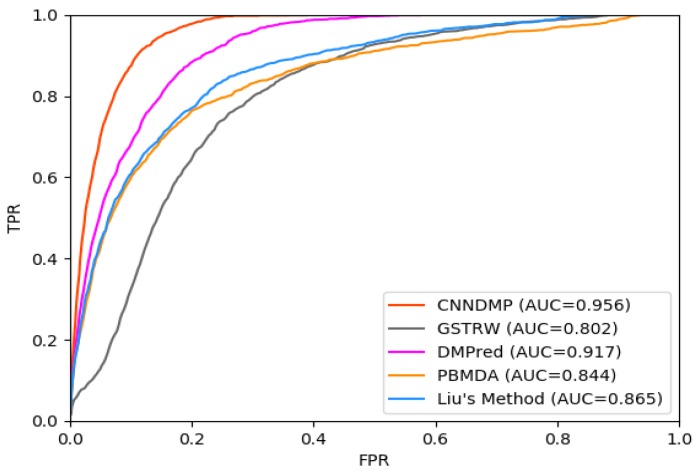
Receiver operating feature curve (ROC) curve of CNNDMP and the other four methods. AUC = area under the curve.

The AUC-ROC values of the five methods (CNNDMP, GSTRW, DMPred, PBMDA, and Liu’s Method) for 15 diseases are 0.956, 0.802, 0.917, 0.844, and 0.865, respectively (Table 2, Figure 1). CNNDMP achieved the best prediction performance, and its average AUC-ROC is 0.956, which is higher by 15.4%, 3.9%, 11.2%, and 9.1% compared to the other four methods, respectively. The miRNA–disease association scores of GSTRW are dependent on the calculation of miRNA similarities and disease similarities. Therefore, GSTRW performs the worst in all methods. The performance of PBMDA is similar to that of Liu’s Method as they all exploit the network topology information. DMPred utilizes miRNA- and disease-related information and achieves a competitive predictive performance. Our method, CNNDMP, completely integrates the original feature of miRNAs, diseases and network topology, combines them with the powerful representation learning capability of CNN and achieves the best prediction performance.

**Figure 2:**
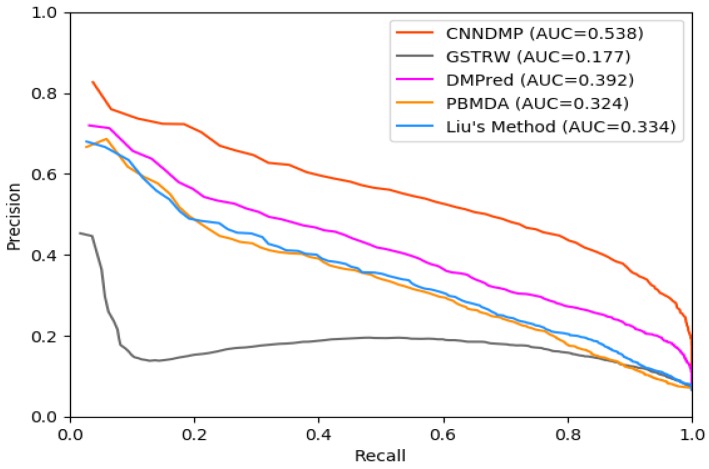
Precision–recall (PR) curve of CNNDMP and the other four methods.

There are far more unobserved miRNA–disease associations than known ones, so there is a serious class imbalance between them. For the imbalanced associations, the PR curves are better than ROC curves in reflecting the prediction performance of different methods. Figure 2 shows the PR curves of CNNDMP, GSTRW, DMPred, PBMDA and Liu’s Method for 15 diseases. Their PR-AUCs are 0.538, 0.177, 0.392, 0.324, and 0.334, respectively. The PR-AUC of CNNDMP is 36.1%, 14.6%, 21.4%, and 20.4% higher than the other methods. As shown in Table 3, CNNDMP yields the best average performance in terms of PR-AUCs and achieves the best performance for 14 of 15 common diseases.

**Figure 3:**
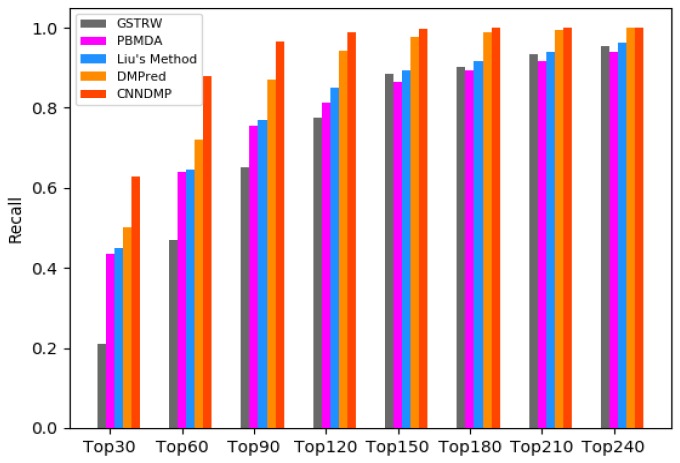
Recall values of top *k* candidates of CNNDMP and the other four methods.

For the top *k* miRNA candidates, the higher recall rate means that there are more positive samples successfully identified. Figure 3 shows the average recall rates for 15 diseases in the top *k* miRNA candidates. CNNDMP’s recall rates for the top 30 to 240 candidate results are 0.629, 0.878, 0.966, 0.990, 0.998, 0.999, 1.0, and 1.0, respectively. The results in Figure 1, Figure 2 and Figure 3 and Table 2 and Table 3 show that our method is indeed effective in discovering potential disease miRNAs.

In addition, to further verify that the ROC-AUC and PR-AUC of CNNDMP are significantly higher than the other methods, we performed a paired *t*-test. All paired *t*-test results are less than 0.05, which indicates that CNNDMP’s performance is significantly better than the other methods (Table 4).

### 2.3. Comparison between the Individual Networks and the Integrated Network

To verify that the performance of the integrated network is better than the individual networks, we evaluate the prediction performances of the left and right networks within CNNDMP, respectively. The values of ROC-AUC and PR-AUC of the left network are 0.916 and 0.509, respectively. For the right network, the values of ROC-AUC and PR-AUC are 0.905 and 0.494, respectively. Compared with the left and right networks, the ROC-AUC of the integrated network increased by 4% and 5.1%, and the PR-AUC increased by 2.9% and 4.4%.

### 2.4. Case Studies on Breast Cancer, Colorectal Cancer and Lung Cancer

To further demonstrate CNNDMP’s ability to discover potential disease-associated miRNAs, we used three independent databases, dbDEMC [31], miRCancer [32], and PhenomiR [33], as well as the relevant literature to verify the candidates of breast cancer, colorectal cancer and lung cancer. We take the prediction results of breast cancer as an example, and list the results of this case analysis in detail.

We list the case study of the top 50 miRNA candidates related to breast cancer in Table 5. dbDEMC is a database of differentially expressed miRNAs in human cancers, and it contains 2224 differentially expressed miRNAs in 36 cancer types. Forty-three of the 50 miRNA candidates are included in this database, which confirmed the differential expression of these candidates in breast cancer. PhenomiR is also a database of differentially expressed miRNAs in human cancers. miRCancer is a miRNA–cancer associations database that collects 6323 miRNA–cancer associations from 4875 academic papers covering 184 cancers. PhenomiR includes two miRNA candidates, and miRCancer contains two candidates. Five miRNA candidates are confirmed in the relevant literature.

The top 50 colorectal cancer-related candidates are given in Supplementary Table S1. The databases of dbDEMC and miRCancer respectively include 48 candidates and one candidate whose abnormal expressions have been identified in colorectal cancer. A candidate marked ‘Unconfirmed’ means that it is not currently supported by the databases and the relevant literature.

In terms of lung cancer, the top 50 candidates are listed in Supplementary Table S2. Forty candidates are included in dbDEMC and three candidates are contained by miRCancer which have abnormal expression in lung cancer. A candidate is supported by PhenomiR to have abnormal regulation in lung cancer. Four candidates are supported by the relevant literature to be differentially expressed in lung cancer. Three candidates marked ‘Unconfirmed’ are not currently supported by the databases and the relevant literature. The case studies on the three diseases confirm that the CNNDMP has a powerful ability to discover potential disease miRNAs.

### 2.5. Predicting Novel Disease-Related miRNAs

By comparing the ROC curve, PR curve and the recall rate of the top *k* candidates for the five methods by cross-validation, CNNDMP has achieved the best prediction performance. Subsequent case analysis results further confirm that CNNDMP has good prediction performance in discovering the associations between miRNAs and diseases. Therefore, we further apply this method to all 326 diseases. We take all the positive samples and the corresponding negative samples as training data. Finally, the top 100 miRNA candidates for each disease are given in Supplementary Table S3.

## 3. Materials and Methods

### 3.1. Dataset

The miRNA–disease associations used in this study derive from the human miRNA–disease database (HMDD) [39]. HMDD has collected thousands of reliable association pairs between miRNAs and diseases. After integrating different miRNA records and unifying the miRNA and disease names, we finally retained 5088 miRNA–disease associations, involving 490 miRNAs and 326 diseases. Disease terms are available from the National Library of Medicine (http://www.ncbi.nlm.nih.gov/mesh). The phenotypic similarities and the semantic similarities are obtained from a published study [18].

### 3.2. Construction of a miRNA–Disease Heterogeneous Network

miRNA similarity measurement. Based on the biological observation that miRNAs with similar functions usually tend to be associated with similar diseases, the similarity of two miRNAs is estimated by measuring the similarities of their associated diseases. For example, miRNA $m_{a}$ is associated with diseases $d_{1}$, $d_{3}$, $d_{5}$, $d_{6}$, and $d_{7}$, whereas miRNA $m_{b}$ is associated with diseases $d_{2}$, $d_{3}$, $d_{4}$, and $d_{6}$. Wang et al. [40] calculated the similarity between $S_{a}=\left\{d_{1},d_{3},d_{5},d_{6},d_{7}\right\}$ and $S_{b}=\left\{d_{2},d_{3},d_{4},d_{6}\right\}$ as the similarity of $m_{a}$ and $m_{b}$, denoted as $M\left(m_{a},m_{b}\right)$. The similarity between $S_{a}$ and $S_{b}$ includes the following three steps: first, the similarities between $d_{1}$ and each of the diseases in $S_{b}$ are calculated, and the maximum similarity is taken as the similarity between $d_{1}$ and $S_{b}$. Similarly, the similarities between $d_{3},d_{5},d_{6},d_{7}$ and $S_{b}$ are obtained, respectively. Second, the similarities between each of diseases in $S_{b}$ and $S_{a}$ are calculated. Finally, these similarities are accumulated and divided by the total number of diseases in $S_{a}$ and $S_{b}$. We use the matrix $M\in R^{N_{m}\times N_{m}}$ to represent the similarities of miRNAs, where $N_{m}$ is the number of miRNAs. The values of miRNA similarities are distributed between 0 and 1.

Disease similarity measurement. The disease similarity measures how similar they are from the perspectives of disease semantics and phenotype. The terms related to a disease are represented by a directed acyclic graph (DAG). If there are more common terms between the DAGs of two diseases, it means that the two diseases are more similar. At the same time, two diseases that share more common phenotypes are often more similar. Therefore, we quantify the similarity of two diseases based on their semantics and phenotype. Xuan et al. have successfully integrated this information and calculated the similarities between diseases. Therefore, disease similarities can be obtained from published studies [19,41]. We use the matrix $D\in R^{N_{d}\times N_{d}}$ to represent the similarities between diseases and values of the similarities vary from 0 and 1, where $N_{d}$ represents the number of diseases.

miRNA–disease associations. If miRNA $m_{i}$ is associated with disease $d_{j}$ then $A_{ij}=1$, or $A_{ij}=0$ when their association has not been observed. We use $A\in R^{N_{m}\times N_{d}}$ to represent the associations between miRNAs and diseases.

**Figure 4:**
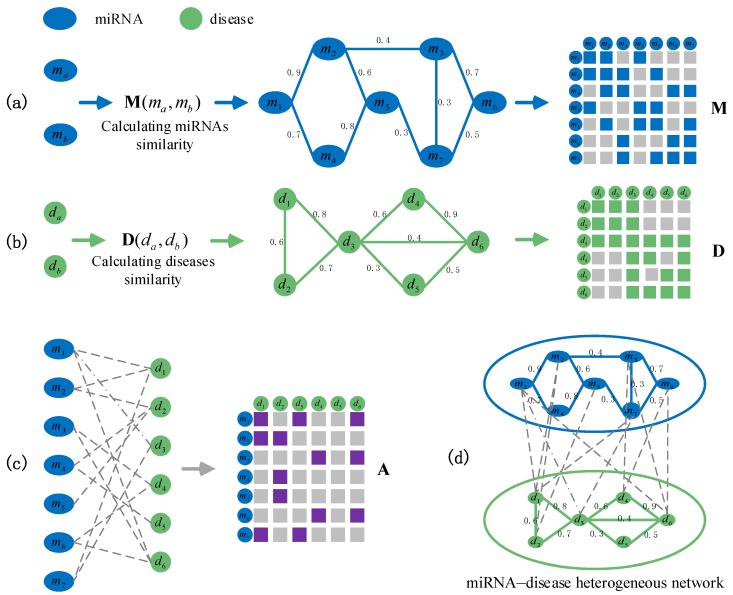
Construction of a miRNA–disease heterogeneous network and matrix representation. (**a**) The miRNA similarities network is constructed based on two miRNAs whose similarity are greater than 0 and the matrix representation M. We represent miRNA network topology information and the similarity values between miRNAs by a weighted network. Each node represents a miRNA entity, and the weight on edge represents miRNA similarity values in the weighted network. (**b**) The disease similarities network and its matrix representation D. (**c**) The miRNA–disease associations network is constructed based on the known associations between miRNAs and diseases, and its corresponding matrix representation A. When a disease is associated with a miRNA, they are connected by a dotted line. (**d**) miRNA–disease heterogeneous network. It effectively integrates miRNA similarities, disease similarities and miRNA–disease association information.

By exploiting the similarities of miRNAs and diseases, as well as the known associations between miRNAs and diseases, we construct a heterogeneous network including two kinds of nodes (miRNAs and diseases), and the matrix representation of the network (Figure 4).

### 3.3. Prediction Model Based on Dual CNN

We construct a prediction model based on dual CNN, which is composed of left and right parts. The left part learns from the original feature information of miRNAs and diseases. The complex, implicit and nonlinear miRNA–disease feature information is captured by the CNN layer. The right part combines miRNA and disease network topology information and represents it deeply by the CNN layer. Finally, we integrate the results of the left and right to obtain final prediction scores for disease-associated miRNAs.

#### 3.3.1. Embedding Layer

**Figure 5:**
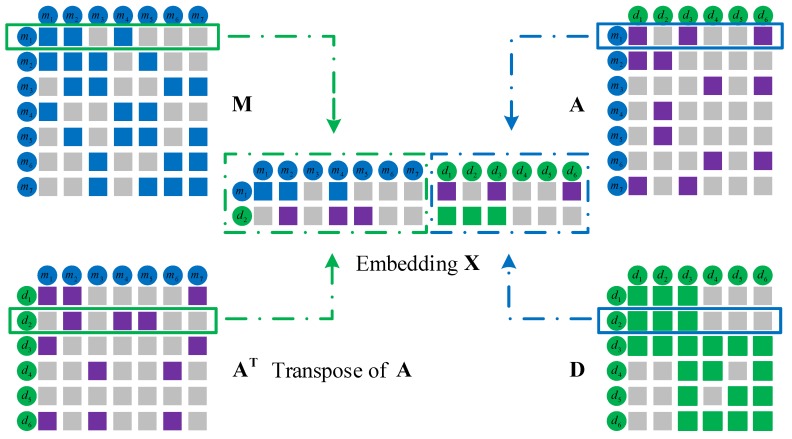
Integration of miRNA and disease original features to construct the embedding in the left part.

Embedding in the left part by integrating miRNA and disease original feature information. Functionally similar miRNAs are usually involved in similar diseases and vice versa. Therefore, we integrate miRNA and disease similarities and miRNA–disease associations to construct the embedding in the left part. We take the miRNA $m_{1}$ and disease $d_{2}$ in Figure 5 as an example to elaborate the integration process. The first row of M represents the similarities between $m_{1}$ and all the miRNAs, and the second row of $A^{T}$ represents the associations between $d_{2}$ and all the miRNAs. The miRNA $m_{1}$ is similar to $m_{2}$ and $m_{4}$, and the disease $d_{2}$ has a known association with $m_{2}$, $m_{4}$ and $m_{5}$. Thus, miRNA $m_{1}$ and disease $d_{2}$ are likely to be associated. Similarly, we integrate the first row of A with the second row of D. Among them, miRNA $m_{1}$ is associated with $d_{1}$, $d_{3}$ and $d_{6}$, and disease $d_{2}$ is similar to $d_{1}$ and $d_{3}$, so miRNA $m_{1}$ and disease $d_{2}$ are likely to be associated. The final integration result is represented by the feature matrix $X\in R^{2\times \left(N_{m}+N_{d}\right)}$.

Embedding in the right part by integrating the networks topology. We firstly obtain network topology information by random walking in the miRNA and disease networks, respectively. The basic principle of a random walk with restart is that the walker starts from a node in the network at 0th time and walks randomly in the miRNA (or disease) network. When the current node of the walker is more similar to a neighbor node, the probability that the walker turns to it is greater. Therefore, after the walking process converges, the probability that the walker reaches a certain node is greater, indicating that the node is more similar to the starting node. We define the convergent vector as $p^{\infty }$, which represents the similarities between the starting node and all the nodes.

We take the miRNA network as an example to illustrate its computational process in detail. Firstly, we need to row-normalize the original miRNA similarities matrix **M** to obtain the probabilistic transfer matrix **W**. Then, based on the following random walk with restart iteration formula,

(3) $$p\left(t+1\right)=\left(1-\beta \right)W^{T}p\left(t\right)+\beta p\left(0\right)$$

the network topology-based miRNA similarities are obtained. Taking miRNA $m_{1}$ as an example, the current random walk from node $m_{1}$, the first element of p(0) is then set to 1 and the other elements are 0. The parameter β∈(0,1) represents the probability that the walker returns to the starting node $m_{1}$ for re-walking. $W^{T}$ is the transposed matrix of W, p(t) represents the probability that the walker arrives at each miRNA node at time t, and p(t+1) represents the arrival probability at time t+1. After the walking process is converged, the vector $p_{m_{1}}^{\infty }$ is obtained and regarded as a part of the embedding in the right part. When $L_{1}$ norm between p(t+1) and p(t) is less than $10^{-6}$, the convergence condition is satisfied. Similarly, in the disease network, we randomly walk from the disease $d_{2}$ node, and finally get the vector $p_{d_{2}}^{\infty }$ as a part of the right embedding.

**Figure 6:**
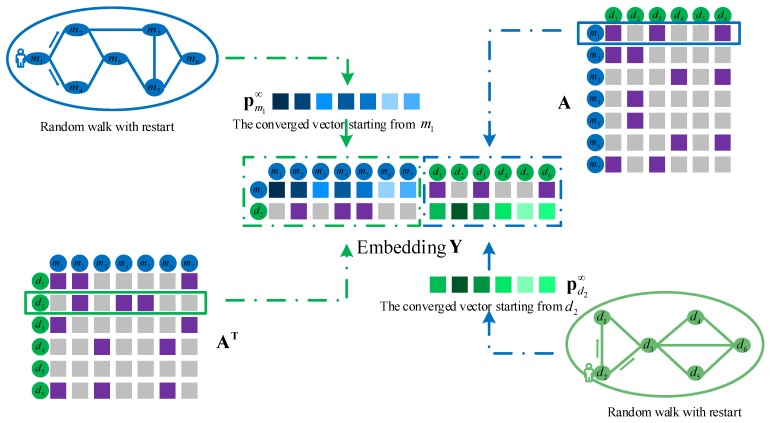
Integration of miRNA and disease network topological features to construct the embedding in the right part.

We integrate the similarity and association information of miRNA $m_{1}$ and disease $d_{2}$ based on network topology to form the embedding in Figure 6. The final integration result is represented by the feature matrix $Y\in R^{2\times \left(N_{m}+N_{d}\right)}$.

#### 3.3.2. Convolutional Module on the Left

**Figure 7:**
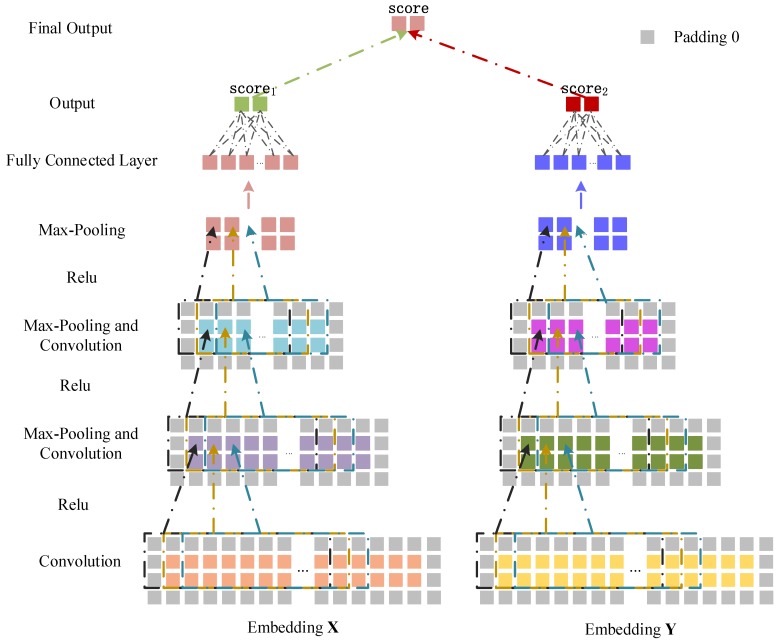
miRNA–disease association prediction framework based on dual CNN.

We treat the embedding $X\in R^{2\times \left(N_{m}+N_{d}\right)}$ as the input data of the CNN module to learn the original feature representation (Figure 7). For the convolutional layer, we set the length and width of a convolution filter to $w_{f}$ and d, and the $n_{conv}$ convolution filters can be represented as $W_{conv}\in R^{w_{f}\times d\times n_{conv}}$. We apply $W_{conv}$ to X to get the feature output $Z_{1}\in R^{2\times \left(N_{m}+N_{d}-w_{f}+1\right)\times n_{conv}}$,

(4) $$X_{conv,i}=\left(X_{i1},X_{i2},\ldots ,X_{i\left(i1+w_{f}-1\right)}\right),X_{conv,i}\in R^{W_{f}\times d}$$

(5) $$\begin{array}{l} Z_{1}\left(2,i,j\right)=g\left(X_{conv,i}*W_{conv}\left(:,:,j\right)+b_{conv}\left(j\right)\right)\\ i\in \left[1,N_{m}+N_{d}-w_{f}+1\right],j\in \left[1,n_{conv}\right] \end{array}$$

where $Z_{1}\left(2,i,j\right)$ is the convolution result when the *j*th convolution filter slides to the *i*th position of X, and g is a nonlinear activation function (relu). $b_{conv}$ is a bias vector, and $X_{i1}$ is the first column vector in the sliding window when the filter moves to the *i*th position of X. In the pooling layer, the max-pooling operation is performed on $Z_{1}$ to get $Q_{1}\in R^{2\times \frac{1}{2}\left(N_{m}+N_{d}\right)\times n_{conv}}$,

(6) $$Q_{1}\left(2,p,j\right)=\max\left(Z_{1}\left(2,r,j\right),Z_{1}\left(2,r+w_{p}-1,j\right)\right)$$

where $Q_{1}\left(2,p,j\right)$ is the pooling value of the *p*th position of the *j*th convolution filter and $w_{p}$ is the sliding window length of the pooling operation. We use $Q_{1}$ as the input of the second convolutional layer, and obtain the output of the second pooling layer $Q_{2}\in R^{2\times \frac{1}{4}\left(N_{m}+N_{d}\right)\times 2n_{conv}}$. Similarly, $Q_{2}$ as the input of the third convolutional layer can obtain $Q_{3}\in R^{2\times \frac{1}{8}\left(N_{m}+N_{d}\right)\times 3n_{conv}}$. Finally, we flatten $Q_{3}$ to a column vector $q\in R^{v\times 1}\;\left(v=2\times \frac{1}{8}\left(N_{m}+N_{d}\right)\times 3n_{conv}\right),$ and obtain the association prediction score of $m_{1}$ and $d_{2}$ through the fully connected layer. The score is defined as $score_{1}\in R^{2\times 1}$,

(7) $${\mathrm{score}}_{1}=H\times q$$

where $H\in R^{2\times v}$ is a weight matrix between the fully connected layer and the output layer.

#### 3.3.3. Convolutional Module on the Right

The embedding $Y\in R^{2\times \left(N_{m}+N_{d}\right)}$ in the right part is input to learn the feature representation of the network topology (Figure 7). The convolution and pooling processes of the right part are similar to that in the left part. The convolutional operation $Z_{2}$ and the max-pooling operation $U_{1}$ are defined as follows,

(8) $$Y_{conv,i}=\left(Y_{i1},Y_{i2},\ldots ,Y_{i\left(i1+w_{f}-1\right)}\right),Y_{conv,i}\in R^{w_{f}\times d}$$

(9) $$Z_{2}\left(2,i,j\right)=g\left(Y_{conv,i}*W_{conv}\left(:,:,j\right)+b_{conv}\left(j\right)\right)$$

(10) $$U_{1}\left(2,p,j\right)=\max\left(Z_{2}\left(2,r,j\right),Z_{2}\left(2,r+w_{p}-1,j\right)\right)$$

where $Z_{2}$ is the feature output of the convolution operation and $Y_{i1}$ is the first column vector in the sliding window when the filter moves to the *i*th position of Y. $U_{1}$ is obtained by performing the max-pooling operation on $Z_{2}$. We use $U_{1}$ as the input of the second convolutional layer, and obtain the output of the second pooling layer $U_{2}\in R^{2\times \frac{1}{4}\left(N_{m}+N_{d}\right)\times 2n_{conv}}$. Similarly, $U_{2}$ as the input of the third convolutional layer can obtain $U_{3}\in R^{2\times \frac{1}{8}\left(N_{m}+N_{d}\right)\times 3n_{conv}}$. Finally, we flatten $U_{3}$ to the column vector $p\in R^{v\times 1},\left(v=2\times \frac{1}{8}\left(N_{m}+N_{d}\right)\times 3n_{conv}\right)$ and get the association score between $m_{1}$ and $d_{2}$ by the fully connected layer. The score is defined as $score_{2}\in R^{2\times 1}$,

(11) $${\mathrm{score}}_{2}=K\times p$$

where $K\in R^{2\times v}$ is the weight matrix between the fully connected layer and the output layer.

#### 3.3.4. Combined Strategy

The association scores $score_{1}$ and $score_{2}$ are obtained from different perspectives of miRNA–disease information. To take complete advantage of the prediction results from the left and right parts, we integrate the two scores as the final association score between a miRNA and a disease. It is defined as follows,

(12) $$\mathrm{score}=\lambda \times {\mathrm{score}}_{1}+\left(1-\lambda \right)\times {\mathrm{score}}_{2}$$

where the parameter λ∈(0,1) is used to adjust the importance of $score_{1}$ and $score_{2}$. The loss functions of the left and right CNNs are defined as $loss_{1}$ and $loss_{2},$

(13) $$loss_{1}=-\overset{T}{\underset{i=1}{\sum }}\left[y_{label}\times \log a+\left(1-y_{label}\right)\times \log\left(1-a\right)\right]$$

(14) $$a=\frac{e^{score_{1}\left(2\right)}}{\sum_{j=1}^{2}e^{score_{1}\left(j\right)}}$$

(15) $$loss_{2}=-\overset{T}{\underset{i=1}{\sum }}\left[y_{label}\times \log b+\left(1-y_{label}\right)\times \log\left(1-b\right)\right]$$

(16) $$b=\frac{e^{score_{2}\left(2\right)}}{\sum_{j=1}^{2}e^{score_{2}\left(j\right)}}$$

where $y_{label}$ indicates the actual association between a miRNA and a disease. $y_{label}$ is 1 when the miRNA is associated with the disease, otherwise $y_{label}$ is 0. $score_{1}\left(1\right)$ and $score_{1}\left(2\right)$ represent the scores of miRNA–disease associations that are classified as the negative sample and the positive one, respectively. a and b indicate the corresponding probabilities obtained by the softmax function. T represents the number of training samples.

## 4. Conclusions

A novel method based on a dual convolutional neural network, CNNDMP, is developed for prioritizing potential disease miRNAs. CNNDMP’s embedding layer is constructed from the biological perspective by combining the biological premise about miRNA–disease associations. At the same time, the embedding layer captures the original similarities and associations of miRNAs and diseases, as well as the topology structure of the miRNA and disease networks. The new framework based on a dual convolutional neural network is constructed for learning the deep features of the original similarities and associations of miRNAs and diseases, and the new miRNA and disease similarities. The results of cross-validation on 15 common diseases confirms CNNDMP’s superior performance. The case studies on three diseases further show that CNNDMP has a strong ability to discover candidate disease miRNAs.

## Figures and Tables

**Table 1:** ROC-AUCs and PR-AUCs at different values of λ.

Parameter λ	0.1	0.2	0.3	0.4	0.4	0.5	0.7	0.8	0.9
ROC-AUC	0.890	0.918	0.934	0.939	0.946	0.950	0.952	0.954	0.956
PR-AUC	0.340	0.401	0.442	0.462	0.491	0.503	0.513	0.521	0.538

**Table 2:** Prediction results of CNNDMP and the other four methods for 15 diseases in terms of ROC-AUCs.

Disease Name	ROC-AUC CNNDMP	GSTRW	DMPred	PBMDA	Liu’s Method
Breast neoplasm	0.987	0.822	0.938	0.852	0.863
Hepatocellular carcinoma	0.986	0.779	0.900	0.803	0.845
Renal cell carcinoma	0.950	0.816	0.903	0.813	0.832
Squamous cell carcinoma	0.936	0.817	0.908	0.881	0.890
Colorectal neoplasm	0.910	0.737	0.842	0.826	0.857
Glioblastoma	0.926	0.814	0.904	0.803	0.842
Heart failure	0.972	0.817	0.987	0.791	0.828
Acute myeloid leukemia	0.961	0.788	0.890	0.844	0.874
Lung neoplasm	0.962	0.791	0.948	0.905	0.920
Melanoma	0.978	0.789	0.913	0.836	0.860
Ovarian neoplasm	0.958	0.830	0.929	0.889	0.897
Pancreatic neoplasm	0.945	0.838	0.916	0.891	0.904
Prostatic neoplasm	0.964	0.822	0.951	0.843	0.855
Stomach neoplasm	0.954	0.762	0.908	0.821	0.836
Urinary bladder neoplasm	0.956	0.816	0.919	0.854	0.865
Average AUC	0.956	0.802	0.917	0.844	0.865

**Table 3:** Prediction results of CNNDMP and the other four methods for 15 diseases in terms of PR-AUCs.

Diseases Name	PR-AUC CNNDMP	GSTRW	DMPred	PBMDA	Liu’s Method
Breast neoplasm	0.894	0.322	0.699	0.574	0.573
Hepatocellular carcinoma	0.893	0.279	0.501	0.454	0.498
Renal cell carcinoma	0.365	0.150	0.293	0.181	0.186
Squamous cell carcinoma	0.287	0.109	0.213	0.211	0.208
Colorectal neoplasm	0.367	0.141	0.186	0.367	0.371
Glioblastoma	0.330	0.151	0.219	0.217	0.243
Heart failure	0.602	0.191	0.700	0.168	0.189
Acute myeloid leukemia	0.368	0.140	0.211	0.191	0.236
Lung neoplasms	0.636	0.147	0.511	0.537	0.503
Melanoma	0.657	0.171	0.389	0.363	0.397
Ovarian neoplasm	0.490	0.169	0.404	0.361	0.361
Pancreatic neoplasm	0.555	0.137	0.329	0.364	0.354
Prostatic neoplasm	0.568	0.166	0.463	0.282	0.264
Stomach neoplasm	0.608	0.220	0.446	0.344	0.346
Urinary bladder neoplasm	0.470	0.163	0.315	0.252	0.280
Average AUC	0.538	0.177	0.392	0.324	0.334

**Table 4:** Comparison of different methods based on AUCs with a paired *t*-test.

*p*-Value	DMPred	GSTRW	PBMDA	Liu’s Method
*p*-value of ROC-AUC between CNNDMP and other methods	6.44998 × 10^−4^	9.60973 × 10^−16^	2.65553 × 10^−10^	1.25344 × 10^−10^
*p*-value of PR-AUC between CNNDMP and other methods	0.02972	1.75747 × 10^−6^	0.00111	0.00151

**Table 5:** The top 50 breast cancer-related candidates.

Rank	miRNA Name	Evidence	Rank	miRNA Name	Evidence
1	hsa-mir-1266	dbDEMC	26	hsa-mir-663	dbDEMC
2	hsa-mir-942	dbDEMC	27	hsa-mir-545	dbDEMC
3	hsa-mir-384	dbDEMC	28	hsa-mir-525	dbDEMC
4	hsa-mir-374b	dbDEMC	29	hsa-mir-520f	dbDEMC
5	hsa-mir-1293	dbDEMC	30	hsa-mir-520g	dbDEMC
6	hsa-mir-3148	Literature [34]	31	hsa-mir-659	dbDEMC
7	hsa-mir-569	Literature [35]	32	hsa-mir-150	miRCancer, PhenomiR
8	hsa-mir-431	dbDEMC	33	hsa-mir-592	dbDEMC
9	hsa-mir-711	Literature [36]	34	hsa-mir-1254	dbDEMC
10	hsa-mir-325	dbDEMC	35	hsa-mir-548c	dbDEMC
11	hsa-mir-1302	Literature [37]	36	hsa-mir-675	miRCancer
12	hsa-mir-33a	dbDEMC	37	hsa-mir-3940	Literature [38]
13	hsa-mir-1246	dbDEMC	38	hsa-mir-1299	dbDEMC
14	hsa-mir-376b	dbDEMC	39	hsa-mir-377	dbDEMC
15	hsa-mir-487a	dbDEMC	40	hsa-mir-519a	dbDEMC
16	hsa-mir-1236	dbDEMC	41	hsa-mir-1180	dbDEMC
17	hsa-mir-548a	dbDEMC	42	hsa-mir-1184	dbDEMC
18	hsa-mir-624	dbDEMC	43	hsa-mir-3151	dbDEMC
19	hsa-mir-633	dbDEMC	44	hsa-mir-627	dbDEMC
20	hsa-mir-1181	dbDEMC	45	hsa-mir-1273a	dbDEMC
21	hsa-mir-382	dbDEMC	46	hsa-mir-1972	dbDEMC
22	hsa-mir-448	dbDEMC	47	hsa-mir-208a	dbDEMC, PhenomiR
23	hsa-mir-583	dbDEMC	48	hsa-mir-668	dbDEMC
24	hsa-mir-518a	dbDEMC	49	hsa-mir-635	dbDEMC
25	hsa-mir-433	dbDEMC	50	hsa-mir-619	dbDEMC

## Supplementary Materials

The following are available online at http://www.mdpi.com/1422-0067/19/12/3732/s1.
