# Supplementary material for: Dual Convolutional Neural Network Based Method for Predicting Disease-Related miRNAs
Source: Int J Mol Sci. 2018 Nov 23;19(12):3732. doi: 10.3390/ijms19123732 (PMC6321160; doi:10.3390/ijms19123732)
Supplement: Supplementary file 1 [file ijms-19-03732-s001.zip › Table S1_colorectal_cancer.docx]

**Supplementary Table S1**

**The top 50** **colorectal cancer-related miRNA candidates** (1) By analyzing the results of biological experiments, ‘dbDEMC’ showed abnormal expression of miRNA in colorectal cancer compared with normal tissues. (2) ‘miRCancer’ means an association between a miRNA and colorectal cancer was included by the database miRCancer. (3) ‘Unconfirmed’ means that this potential miRNA candidate is not currently supported by databases and literature.

| Rank | MiRNA Name | Evidence | Rank | MiRNA Name | Evidence |
| --- | --- | --- | --- | --- | --- |
| 1 | hsa-mir-668 | dbDEMC | 26 | hsa-mir-1293 | dbDEMC |
| 2 | hsa-mir-548a | dbDEMC | 27 | hsa-mir-711 | dbDEMC |
| 3 | hsa-mir-3148 | dbDEMC | 28 | hsa-mir-384 | dbDEMC |
| 4 | hsa-mir-569 | dbDEMC | 29 | hsa-mir-1266 | dbDEMC |
| 5 | hsa-mir-592 | dbDEMC | 30 | hsa-mir-525 | dbDEMC |
| 6 | hsa-mir-663 | dbDEMC | 31 | hsa-mir-548c | dbDEMC |
| 7 | hsa-mir-1236 | dbDEMC | 32 | hsa-mir-376b | dbDEMC |
| 8 | hsa-mir-545 | dbDEMC | 33 | hsa-mir-659 | dbDEMC |
| 9 | hsa-mir-622 | dbDEMC | 34 | hsa-mir-624 | dbDEMC |
| 10 | hsa-mir-564 | dbDEMC | 35 | hsa-mir-523 | dbDEMC |
| 11 | hsa-mir-648 | dbDEMC | 36 | hsa-mir-942 | dbDEMC |
| 12 | hsa-mir-941 | dbDEMC | 37 | hsa-mir-3151 | dbDEMC |
| 13 | hsa-mir-369 | dbDEMC | 38 | hsa-mir-1207 | dbDEMC |
| 14 | hsa-mir-1302 | dbDEMC | 39 | hsa-mir-188 | dbDEMC |
| 15 | hsa-mir-561 | dbDEMC | 40 | hsa-mir-630 | dbDEMC |
| 16 | hsa-mir-614 | dbDEMC | 41 | hsa-mir-675 | dbDEMC |
| 17 | hsa-mir-1181 | dbDEMC | 42 | hsa-mir-518c | dbDEMC |
| 18 | hsa-mir-599 | dbDEMC | 43 | hsa-mir-671 | dbDEMC |
| 19 | hsa-mir-448 | dbDEMC | 44 | hsa-mir-487a | dbDEMC |
| 20 | hsa-mir-3179 | dbDEMC | 45 | hsa-mir-662 | dbDEMC |
| 21 | hsa-mir-550b | dbDEMC | 46 | hsa-mir-611 | dbDEMC |
| 22 | hsa-mir-720 | dbDEMC | 47 | hsa-mir-600 | dbDEMC |
| 23 | hsa-mir-583 | dbDEMC | 48 | hsa-mir-619 | dbDEMC |
| 24 | hsa-mir-325 | dbDEMC | 49 | hsa-mir-582 | miRCancer |
| 25 | hsa-mir-374b | dbDEMC | 50 | hsa-mir-1972 | Unconfirmed |
