# Supplementary material for: Dual Convolutional Neural Network Based Method for Predicting Disease-Related miRNAs
Source: Int J Mol Sci. 2018 Nov 23;19(12):3732. doi: 10.3390/ijms19123732 (PMC6321160; doi:10.3390/ijms19123732)
Supplement: Supplementary file 1 [file ijms-19-03732-s001.zip › Table S2_lung_cancer.docx]

**Supplementary Table S2**

**The top 50 lung cancer-related miRNA candidates** (1) By analyzing the results of biological experiments, ‘dbDEMC’ showed abnormal expression of miRNA in lung cancer compared with normal tissues. (2) ‘miRCancer’ means an association between a miRNA and breast cancer was included by the database miRCancer. (3) ‘PhenomiR’ is also a database of differentially expressed miRNAs in lung cancers by analyzing the results of microarray experiments and PCR experiments. (4) ‘Literature’ means that there is a published literature to support that a miRNA is upregulated or downregulated in lung cancer. (5) ‘Unconfirmed’ means that this potential miRNA candidate is not currently supported by databases and literature.

| Rank | MiRNA Name | Evidence | Rank | MiRNA Name | Evidence |
| --- | --- | --- | --- | --- | --- |
| 1 | hsa-mir-384 | miRCancer | 26 | hsa-mir-1207 | miRCancer |
| 2 | hsa-mir-668 | dbDEMC | 27 | hsa-mir-599 | dbDEMC |
| 3 | hsa-mir-550b | Literature [1] | 28 | hsa-mir-614 | dbDEMC |
| 4 | hsa-mir-325 | dbDEMC | 29 | hsa-mir-569 | Literature [3] |
| 5 | hsa-mir-3179 | Literature [2] | 30 | hsa-mir-648 | Unconfirmed |
| 6 | hsa-mir-487a | dbDEMC | 31 | hsa-mir-3148 | Literature [4] |
| 7 | hsa-mir-624 | dbDEMC | 32 | hsa-mir-1254 | dbDEMC |
| 8 | hsa-mir-525 | dbDEMC | 33 | hsa-mir-382 | dbDEMC |
| 9 | hsa-mir-659 | dbDEMC | 34 | hsa-mir-3940 | miRCancer |
| 10 | hsa-mir-523 | dbDEMC | 35 | hsa-mir-28 | dbDEMC |
| 11 | hsa-mir-548a | dbDEMC | 36 | hsa-mir-106b | dbDEMC, PhenomiR |
| 12 | hsa-mir-520f | dbDEMC | 37 | hsa-mir-1293 | dbDEMC |
| 13 | hsa-mir-431 | dbDEMC | 38 | hsa-mir-1299 | dbDEMC |
| 14 | hsa-mir-520g | Unconfirmed | 39 | hsa-mir-374b | dbDEMC |
| 15 | hsa-mir-548c | dbDEMC | 40 | hsa-mir-598 | dbDEMC |
| 16 | hsa-mir-377 | dbDEMC | 41 | hsa-mir-519b | dbDEMC |
| 17 | hsa-mir-376b | dbDEMC | 42 | hsa-mir-941 | dbDEMC |
| 18 | hsa-mir-663 | dbDEMC | 43 | hsa-mir-675 | dbDEMC |
| 19 | hsa-mir-448 | dbDEMC | 44 | hsa-mir-633 | dbDEMC |
| 20 | hsa-mir-1181 | dbDEMC | 45 | hsa-mir-329 | dbDEMC |
| 21 | hsa-mir-592 | dbDEMC | 46 | hsa-mir-518a | dbDEMC |
| 22 | hsa-mir-545 | dbDEMC | 47 | hsa-mir-767 | dbDEMC |
| 23 | hsa-mir-1302 | Unconfirmed | 48 | hsa-mir-1266 | dbDEMC |
| 24 | hsa-mir-548b | dbDEMC | 49 | hsa-mir-298 | dbDEMC |
| 25 | hsa-mir-1236 | dbDEMC | 50 | hsa-mir-501 | dbDEMC |

**Reference:**

[1] Parimon T, Brauer R, Schlesinger S Y, et al. Syndecan-1 Controls Lung Tumorigenesis by Regulating miRNAs Packaged in Exosomes[J]. The American journal of pathology, 2018, 188(4): 1094-1103.

[2] Liu M, Zhou K, Cao Y. MicroRNA-944 affects cell growth by targeting EPHA7 in non-small cell lung cancer[J]. International journal of molecular sciences, 2016, 17(10): 1493.

[3] Zheng Y P, Wu L, Gao J, et al. Tumor suppressive role of miR-569 in lung cancer[J]. Oncology letters, 2018, 15(4): 4087-4092.

[4] Ma L, Li P, Wang R, et al. Analysis of novel microRNA targets in drug-sensitive and-insensitive small cell lung cancer cell lines[J]. Oncology reports, 2016, 35(3): 1611-1621.
